# Effect of DLK1 on tumorigenesis in CD34^+^CD38^−^ bone marrow cells in myelodysplastic syndromes

**DOI:** 10.3892/ol.2013.1346

**Published:** 2013-05-14

**Authors:** WEI ZHANG, ZONGHONG SHAO, RONG FU, HUAQUAN WANG, LIJUAN LI, LANZHU YUE

**Affiliations:** Department of Hematology, General Hospital of Tianjin Medical University, Tianjin 300052, P.R. China

**Keywords:** myelodysplastic syndromes, CD34^+^CD38^−^ bone marrow cells, DLK gene, small interfering RNA, tumorigenesis

## Abstract

The myelodysplastic syndromes (MDSs) are a group of clonal stem cell disorders resulting from aberrations within hematopoietic stem cells (HSCs), which may lead to the onset of a number of diseases, including acute myeloid leukemia (AML). Recent studies have demonstrated that the expression levels of the DLK1 gene are increased in MDS. In order to determine whether the addition of DLK1 affects tumorigenesis, small interfering (si)RNAs were designed to target DLK1 in order to knockdown its expression in CD34^+^CD38^−^ bone marrow cells in MDS. A lower proliferative rate was observed in the CD34^+^CD38^−^ bone marrow cells following this knockdown of DLK1 expression. The suppression of DLK1 expression resulted in a less aggressive MDS phenotype, which suggests that the upregulation of DLK1 expression may play an oncogenic role in CD34+CD38^−^ bone marrow cells.

## Introduction

The myelodysplastic syndromes (MDSs) are a heterogeneous group of clonal malignant hematopoietic disorders, which are characterized by ineffective hematopoiesis and a frequent progression to acute myeloid leukemia (AML). Studies suggest that the MDSs are a group of stem-cell disorders in which aberrations within hematopoietic stem cells (HSCs) may lead to the onset of a number of diseases, including AML (1,2). Previous data have confirmed that human AML stem cells reside within the CD34^+^CD38^−^ compartment of the leukemic clone, which is also observed in normal HSCs.

DLK1 is a transmembrane protein of the epidermal growth factor (EGF)-like family that mainly functions as a GF to maintain proliferating cells in an undifferentiated state (3). DLK1, also known as preadipocyte factor-1 (pref-1), fetal antigen 1 (FA1), pG2 and ZOG, is expressed extensively in immature cells and downregulated during fetal development (4), thus suggesting that DLK1 is important in stem/progenitor cells. In hematopoiesis, DLK1 is important in supporting the proliferation of early progenitor cells (5). The overexpression of DLK1 has been observed in numerous types of cancer, including MDS and AML (6,7). Evidence also suggests that DLK1 may inhibit tumor cell differentiation and increase tumorigenic potential (8), although the underlying mechanisms causing these effects remain unclear. Based on this evidence, the present study aimed to examine the hypothesis that DLK1 plays a central role in the tumorigenesis of CD34^+^CD38^−^ cells. This hypothesis represents a novel perspective with regard to the differentiation of CD34^+^CD38^−^ cells induced by the knockdown of DLK1 expression. This study has the potential to shed light on the role of DLK1 in CD34^+^CD38^−^ cells with regard to the regulation of the cell cycle and apoptosis, and to provide mechanistic insights into the progression of malignant tumors.

## Materials and methods

### Patients

A total of 23 untreated patients (10 females, 13 males) who had been newly diagnosed with MDS according to the World Health Organization (WHO) criteria were enrolled in the present study (9). The median age was 56 years (range, 28–76 years). According to the WHO criteria, patients were classified as follows: refractory cytopenia with multilineage dysplasia (RCMD; including RCMD with ringed sideroblasts, RCMD-RS; n=6), refractory anemia with excess blasts-1 (RAEB-1; n=3) and RAEB-2 (n=14). Written informed consent was obtained from each patient prior to entering the trial. The study complied with the acceptable international standards outlined in the Declaration of Helsinki, and was approved by the Institutional Ethics Committee of Tianjin Medical University (Tianjin, China).

### Sampling of bone marrow cells

Bone marrow was obtained from the posterior iliac crest and collected in ethylenediaminetetraacetic acid (EDTA) anticoagulated syringes. Written informed consent was obtained from each patient prior to bone marrow puncture. The bone marrow samples were transferred to the laboratory within 4 h of aspiration.

### Magnetic sorting of CD34^+^CD38^−^ cells

CD34^+^CD38^−^ cells were obtained from the mononuclear cell fraction of the MDS bone marrow samples (Ficoll density gradient separation), followed by immunomagnetic bead selection with monoclonal murine antihuman CD34 and CD38 antibodies using the autoMACS automated separation system (Miltenyi Biotech, Mönchengladbach, Germany). The yield and purity of the positively selected CD34^+^CD38^−^ cells were evaluated by flow cytometry (FACSCalibur; Bio-Rad, Hercules, CA, USA).

### Cell culture and transfection

The CD34^+^CD38^−^ cells were cultured in X-VIVO 10 medium supplemented with 10% fetal bovine serum (FBS) and GFs. The GF cocktail, consisting of 100 ng/ml stem cell factor (Cell Signaling Technology, Inc., Danvers, MA, USA), 100 ng/ml FLT-3 (Reprotech, Rocky Hill, NJ, USA), 20 ng/ml granulocytic CSF (Reprotech), 20 ng/ml IL-3 (Reprotech) and 20 ng/ml IL-6 (Reprotech), was used. All cultures were maintained at 37°C in a moist atmosphere containing 5% CO_2_. The cells were plated in 6-well plates at 2×10^5^ cells/well. Transfection was performed using Lipofectamine 2000 (Invitrogen Life Technologies, Carlsbad, CA, USA) according to the manufacturer’s instructions. The small interfering (si)RNA sequence targeting DLK1 (siRNA-DLK1) was 5′-GCACCUGCGUGGAUGAUGAU UdTdT-3′ and 5′-dTdTAGTAGTAGGTGCGTCCACG-3′. The siRNA sequence for scrambled siRNA (siRNA-scr) was 5′-UUGAAGUUAUGUAUCCUCCUU-3′ and 5′-CUGAAG CUGCUGGGAGUAAUU-3′. Blank transfection served as the control.

### Quantitative (q)PCR assay

Total RNA was prepared using TRIzol (Invitrogen Life Technologies) 48 h after transfection. The total RNA (1 *μ*g) was reverse transcribed into cDNA using AMV reverse transcriptase (Takara Bio, Inc., Osaka, Japan) according to the manufacturer’s instructions. The DLK1 gene was amplified under the following conditions: 50°C for 2 min and 95°C for 10 min, then 40 cycles at 95°C for 15 sec, followed by 60°C for 60 sec. β-actin served as a reference. The following oligonucleotide sequences were used: DLK1 forward, 5′-CTGGACGGTGGCCTCTATGAATG-3′ and reverse, 5′-ATCATCCACGCAGGTGCCTC-3′; and β-actin forward, 5′-CACGATGGAGGGGCCGGACTCATC-3′ and reverse, 5′-TAAAGACCTCTATGCCAACACAGT-3′. Each sample was performed in triplicate and a reverse transcriptase negative control was also tested to exclude any DNA contamination. The expression ratio was calculated as 2^n^, where n is the C_T_ value difference for each patient normalized by the average C_T_ difference of the samples from the control subjects (ΔΔC_T_ method).

### Western blotting

Cell lysates were prepared on ice in radio-immunoprecipitation assay (RIPA) lysis buffer containing 50 mM Tris-Cl at pH 7.5, 150 mM NaCl, 1% Nonidet-P40, 0.5% sodium deoxycholate, 0.1% sodium dodecyl sulfate and 1–2 mM phenylmethylsulfonyl fluoride (PMSF). The protein concentrations were measured using bicinchoninic acid (BCA) assay reagents (Bio-Rad). A total of 100 *μ*g whole cell lysate protein was separated by 10% sodium dodecyl sulfate-polyacrylamide gel electrophoresis (SDS-PAGE), and the separated protein was transferred onto polyvinylidene difluoride (PVDF) membranes. Subsequent to blocking by incubation with 5% BSA in Tris-buffered saline for 1 h at room temperature, the membrane was incubated using a polyclonal antibody against DLK1 (Cell Signaling Technology, Inc.) and an anti-GAPDH antibody (Tianjin XiangTian Technology Co., Ltd., China).

### Flow cytometry

At 48 h post-transfection, the cells were collected and prepared in a suspended solution, then fixed with 250 *μ*l solution A at room temperature for 10 min. Next, the cells were incubated with 200 *μ*l solution B at room temperature for 10 min, followed by solution C for 10 min at 4°C in the dark. The cell cycle was analyzed using FACSCalibur flow cytometry (Bio-Rad).

At 48 h post-transfection, the cells were collected and washed with cold phosphate-buffered saline (PBS). The cells were incubated with 5 *μ*l Annexin V and 5 *μ*l propidium iodide (PI) at room temperature for 15 min in the dark. Cell apoptosis was analyzed using FACSCalibur flow cytometry (Bio-Rad).

### Statistical analysis

SPSS 16.0 (SPSS, Inc., Chicago, IL, USA) was used to perform the statistical analysis. The data are expressed as the mean ± SD. The paired t-test analysis of variance was used to analyze the significance between groups. The least significant difference method of multiple comparisons with parental and control groups was used when the probability for the analysis of variance was statistically significant. P<0.05 was considered to indicate a statistically significant difference.

## Results

### Inhibition of DLK1 expression with siRNA

**Figure 1.**
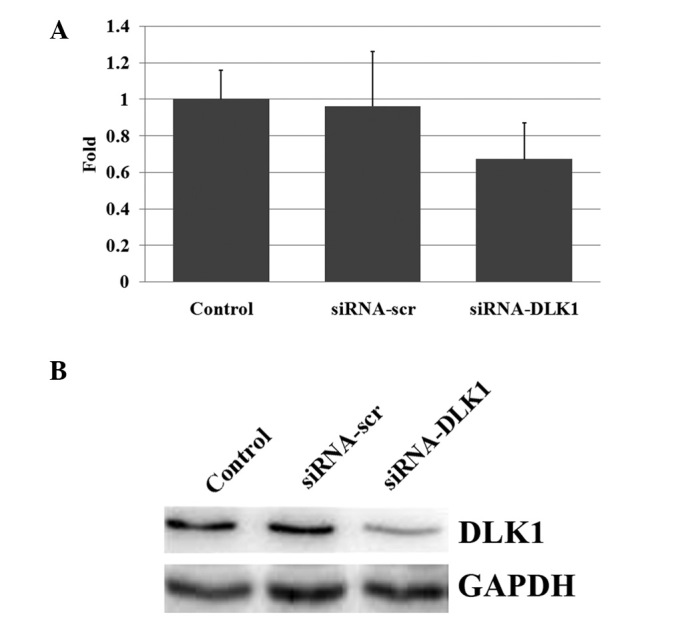
DLK1 expression. (A) DLK1 mRNA expression levels in CD34^+^CD38^−^ cells detected by quantitative (q)PCR. The relative expression of the groups was 1.00 in the control group, 0.93 in the siRNA-scr group and 0.68 in the siRNA-DLK1 group. Error bars represent the SD of 3 replicates used for each group. (B) DLK1 protein expression levels in CD34^+^CD38^−^ cells determined by western blotting. The results were compared with GAPDH. The relative expression of the groups was 1.15 in the control group, 1.23 in the siRNA-scr group and 0.74 in the siRNA-DLK1 group. siRNA-scr, small interfering RNA sequence for scrambled siRNA; siRNA-DLK1, siRNA sequence targeting DLK1.

Following the transfection of the siRNA-DLK1, the mRNA and protein expression levels of DLK1 were inhibited. qPCR amplification revealed significantly decreased levels of DLK1 mRNA expression due to the inhibition caused by the siRNAs (Fig. 1A). A western blot analysis additionally revealed significantly decreased levels of DLK1 protein expression (Fig. 1B). Therefore, DLK1 expression was effectively inhibited by RNA interference.

### Apoptosis in DLK1 knockdown cells

**Figure 2.**
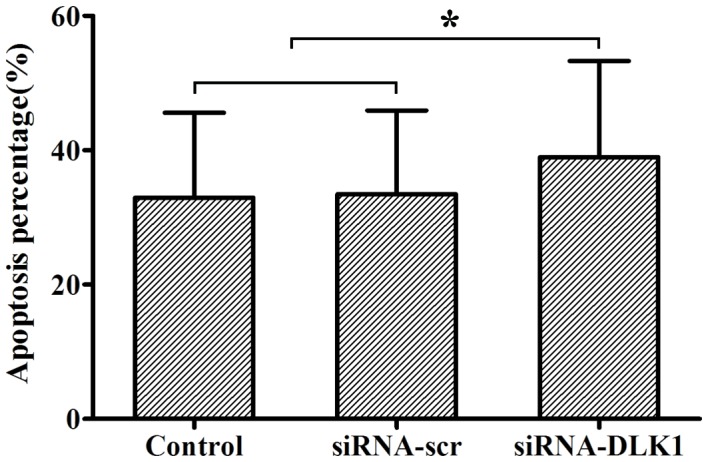
Apoptotic rate in CD34^+^CD38^−^ and DLK1-knockdown cells (%). Significant differences between groups are shown as ^*^P<0.05. siRNA-scr, small interfering RNA sequence for scrambled siRNA; siRNA-DLK1, siRNA sequence targeting DLK1.

The rate of apoptosis was measured by flow cytometry. The apoptotic rate was increased when DLK1 expression was knocked down compared with the siRNA-scr and control groups (38.97±14.32 vs. 33.48±12.44 and 32.94±12.64%, P<0.05; Fig. 2).

### Cell cycle progression in DLK1-knockdown cells

**Figure 3.**
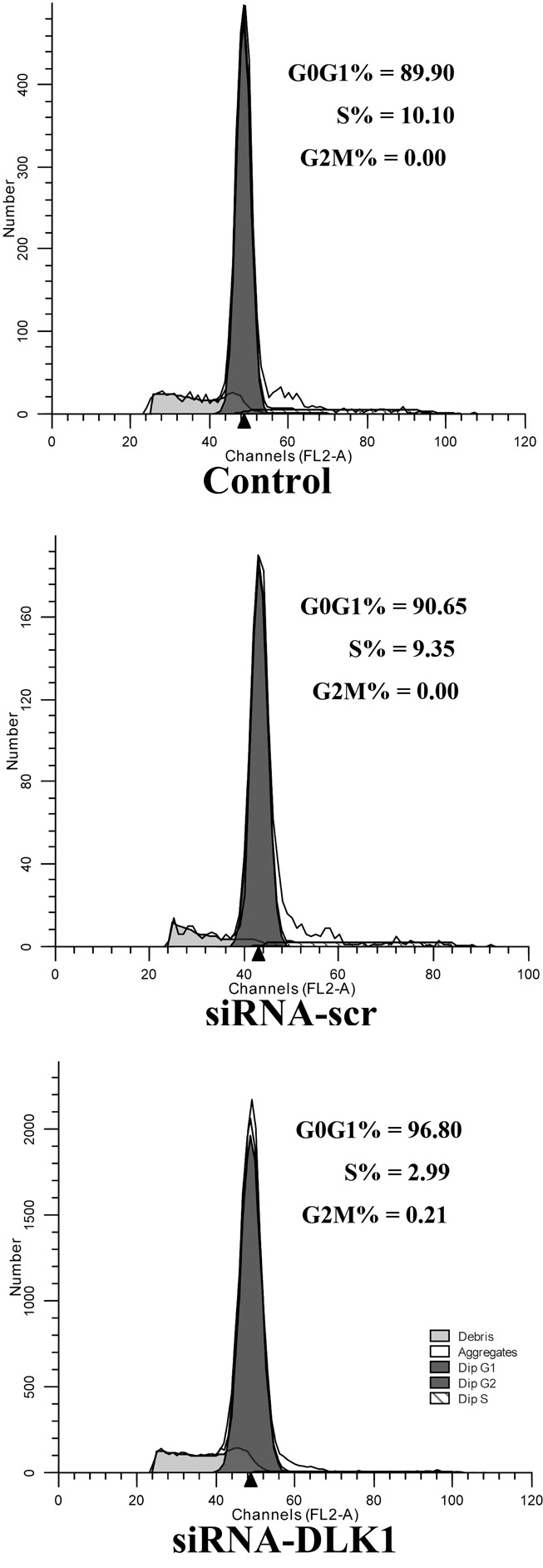
Cell cycle kinetics detected by flow cytometry. Cells from the siRNA-DLK1 group are promoted to S phase. siRNA-scr, small interfering RNA sequence for scrambled siRNA; siRNA-DLK1, siRNA sequence targeting DLK1.

The flow cytometry assay revealed that the cells were stimulated to enter the G_0_/G_1_ phase and inhibited from entering the S phase of the cell cycle when DLK1 expression was knocked down compared with the siRNA-scr and control groups (G_0_/G_1_ phase, 95.81±3.87 vs. 91.29±10.39 and 91.22±10.82%, P<0.05; S phase, 3.90±3.61 vs. 8.41±10.33 and 8.38±10.65%, P<0.05; Fig. 3).

## Discussion

The present study examined the effect of knocking down the expression of DLK1 on tumorigenesis in CD34^+^CD38^−^ cells. DLK1 (pref-1) is a transmembrane and secreted protein, which is a member of the EGF-like family, homologous to Notch/Delta/Serrate. DLK-1 contains a signal peptide followed by 6 EGF-like repeats, a transmembrane domain and a short intracellular tail (3). The DLK1 gene is located within an imprinted region of chromosome 14q32 and expressed only from the paternal allele in normal cells (10). The upregulation of DLK1 has been previously observed in CD34^+^ cells from patients with MDS (11,12). Sakajiri *et al* (6) analyzed hematopoiesis in DLK1-knockout mice and suggested that DLK1 contributed to granulocyte, megakaryocyte and B-cell clonogenic growth, and was required for the generation of splenic B cells.

The elevated expression of DLK1 has been observed in several tumor types, including MDS and AML. However, the role of DLK1 and its mechanism in tumor growth remains to be fully elucidated (13). If DLK1 maintains tumor cells in a stem cell-like state, the relatively long lifespan of stem cells will allow undifferentiated tumor cells to accumulate and the stable genetic and epigenetic changes that ultimately result in tumor malignancy to be perpetuated. The role of DLK1 in tumor progression requires investigating in further detail.

The cell cycle analysis of the present study revealed that DLK1-expressing CD34^+^CD38^−^ cells exhibited a slower progression through the G_0_/G_1_ phase into the S phase compared with the controls. In addition, the apoptotic rate of the CD34^+^CD38^−^ cells was elevated when the expression of DLK1 was inhibited. These findings signify the importance of the DLK1 gene in inhibiting cell differentiation and reducing the rate of cellular apoptosis.

Extremely little is known about the molecular mechanism and signal transduction pathway of DLK1 that is involved in cell differentiation. Thus, these regulatory mechanisms require further investigation.

In conclusion, the present study indicates that the suppression of DLK1 expression in CD34^+^CD38^−^ cells results in a less aggressive phenotype. Impaired differentiation and reduced hematopoietic cell production are important features of hematopoiesis in MDS. These results support the further investigation of the role of DLK1 in abnormal hematopoiesis in MDS.

## References

[1] Corey SJ, Minden MD, Barber DL, Kantarjian H, Wang JC, Schimmer AD (2007). Myelodysplastic syndromes: the complexity of stem-cell diseases. Nat Rev Cancer.

[2] Nimer SD (2008). MDS: a stem cell disorder - but what exactly is wrong with the primitive hematopoietic cells in this disease?. Hematology Am Soc Hematol Educ Program.

[3] Laborda J (2000). The role of the epidermal growth factor-like protein dlk in cell differentiation. Histol Histopathol.

[4] Floridon C, Jensen CH, Thorsen P (2000). Does fetal antigen 1 (FA1) identify cells with regenerative, endocrine and neuroendocrine potentials? A study of FA1 in embryonic, fetal, and placental tissue and in maternal circulation. Differentiation.

[5] Moore KA, Pytowski B, Witte L, Hicklin D, Lemischka IR (1997). Hematopoietic activity of a stromal cell transmembrane protein containing epidermal growth factor-like repeat motifs. Proc Natl Acad Sci USA.

[6] Sakajiri S, O’kelly J, Yin D (2005). DLK1 in normal and abnormal hematopoiesis. Leukemia.

[7] Astuti D, Latif F, Wagner K (2005). Epigenetic alteration at the DLK1-GTL2 imprinted domain in human neoplasia: analysis of neuroblastoma, phaeochromocytoma and Wilms’ tumour. Br J Cancer.

[8] Li L, Forman SJ, Bhatia R (2005). Expression of DLK1 in hematopoietic cells results in inhibition of differentiation and proliferation. Oncogene.

[9] Vardiman JW, Thiele J, Arber DA (2009). The 2008 revision of the World Health Organization (WHO) classification of myeloid neoplasms and acute leukemia: rationale and important changes. Blood.

[10] Khoury H, Suarez-Saiz F, Wu S, Minden MD (2010). An upstream insulator regulates DLK1 imprinting in AML. Blood.

[11] Hofmann WK, de Vos S, Komor M, Hoelzer D, Wachsman W, Koeffler HP (2002). Characterization of gene expression of CD34^+^ cells from normal and myelodysplastic bone marrow. Blood.

[12] Pellagatti A, Cazzola M, Giagounidis A (2006). Gene expression profiles of CD34^+^ cells in myelodysplastic syndromes: involvement of interferon-stimulated genes and correlation to FAB subtype and karyotype. Blood.

[13] Kim Y (2010). Effect of retinoic acid and delta-like 1 homologue (DLK1) on differentiation in neuroblastoma. Nutr Res Pract.

